# Cytokine Levels in Late Pregnancy: Are Female Infants Better Protected Against Inflammation?

**DOI:** 10.3389/fimmu.2015.00318

**Published:** 2015-06-16

**Authors:** Christine Burns, Sharron Therese Hall, Roger Smith, Caroline Blackwell

**Affiliations:** ^1^Hunter Medical Research Institute, New Lambton, NSW, Australia; ^2^Information-Based Medicine, Faculty of Health and Medicine, School of Biomedical Sciences and Pharmacy, University of Newcastle, New Lambton, NSW, Australia; ^3^Hunter Area Pathology Service Immunology, New Lambton, NSW, Australia; ^4^Faculty of Health and Medicine, Mothers and Babies Research Centre, University of Newcastle, New Lambton, NSW, Australia

**Keywords:** cytokines, amniotic fluid, maternal plasma, cord blood, third trimester

## Abstract

Inflammatory responses have been implicated in several forms of infant deaths (sudden expected deaths and stillbirths) and the initiation of pre-term births. In this study, we examined matched samples of term maternal blood, cord blood, and amniotic fluid obtained from 24 elective cesarean deliveries for both pro- and anti-inflammatory cytokines thought to be important in maintaining a balanced response leading to successful pregnancy outcome. These included interleukin (IL)-1β, IL-6, IL-8, tumor necrosis factor-α (TNF-α), interferon-γ (IFN-γ), IL-10, and IL-1 receptor antagonist (IL-1ra). Amniotic fluid levels for each of the cytokines examined were significantly higher than those for cord blood or maternal plasma. While pro-inflammatory cytokines were higher in amniotic fluid associated with male fetuses compared with females, the major significant difference was higher levels of IL-1ra in amniotic fluid associated with female fetuses. Our study supports similar findings for cytokines during mid-trimester, which noted that amniotic fluid levels were higher than those in maternal blood. Our study suggests that maternal decidua secretes additional IL-ra in the presence of a female conceptus which improves the likelihood of a good outcome compared to pregnancies with male fetuses.

## Introduction

A balanced cytokine response is thought to be important in maintaining pregnancy (1). Our previous studies indicated that there were differences in inflammatory responses associated with sex and that testosterone levels present during critical development periods might influence pro-inflammatory responses to infection (2, 3). There is an excess of males among infants who die suddenly and unexpectedly (4, 5), stillbirths (6, 7), and pre-term births (8, 9). As inflammation has been implicated in each of these conditions, we examined cytokine levels in matched sets of samples of maternal plasma, cord blood, and amniotic fluid collected during elective term cesarean deliveries to determine: if in the third trimester, cytokine levels in maternal blood and amniotic fluid were higher than those reported for the second trimester (10); if there was a correlation between cytokine levels in maternal or cord blood or amniotic fluid samples; if there were differences in cytokine levels from samples associated with male and female fetuses.

## Materials and Methods

Matched samples of maternal plasma, cord blood, and amniotic fluid were obtained from elective term cesarean section deliveries as part of a study approved by the Hunter New England Ethics Committee, Mapping the Progress of Human Parturition (02/06/12/3.13). Twenty-four sets of samples were examined, 12 from pregnancies with a female infant and 12 from pregnancies with a male infant. A Bio-Plex suspension array assay (Bio-Rad) was used to quantitate interleukin (IL)-1β, IL-6, IL-8, interferon-γ (IFN-γ), tumor necrosis factor-α (TNF-α), and the anti-inflammatory mediators IL-10 and IL-1 receptor antagonist (IL-1ra) (11).

The samples were stored at −80°C. Specimens were thawed, vortexed, and a 200 μl aliquot filtered with a 0.2 μm centrifugal filter (Millipore). The filtrate was either refrozen at −80°C for later analysis or immediately diluted for cytokine assay in cold BioPlex diluent. Samples from maternal or cord blood were diluted one volume in two volumes and amniotic fluid was diluted one volume in three volumes of Bio-Plex sample diluent.

Results were expressed in pg ml^−1^. Data were tabulated as median and ranges. Spearman’s non-parametric correlations were used to determine relationships between variables. Student’s *t*-test was used on log-transformed data to assess differences in cytokine levels between the three sources of samples and between male and female infants. Significance was set at 5%.

## Results

The first objective of the study was to compare cytokine levels in maternal blood and amniotic fluid with data reported for an Australian population at mid-trimester (Table 1). In the current study, the median gestational age (GA) at time of sampling was 39.1 weeks. In many cord blood and maternal plasma samples in the current study, levels of IFN-γ, TNF-α, and IL-1β were below the lower limits of detection.

**Table 1:** **Comparison of cytokine levels in maternal blood (a) and amniotic fluid (b) at trimester 2 (10) and trimester 3**.

	Maternal blood	
	14–16 weeks *N* = 33	37.6–40.3 weeks *N* = 24
	
	Median (range) (pg ml^−1^) *N* = 33	Median (range) (pg ml^−1^) *N* = 24
IFN-γ	113 (34–431)	41 (< 11 – 772)
IL-1β	7 (2–360)	< 2.4 (< 2.4 – 21)
IL-6	22 (6–2116)	59 (10 – 74)
IL-8	17 (3–98)	90 (36 – 3289)
TNF-α	53 (4–392)	16 (< 5.4 – 32)
IL-10	3 (<1–1206)	11 (< 1.8 – 160)
IL-1ra	284 (21–8620)	90 (< 4 – 230)

	**Amniotic fluid**	
	**14–16 weeks *N* = 100**	**37.6 – 40.3 weeks *N* = 24**
	
	**Median (range) (pg ml^−1^) (*n* = 100)**	**Median (range) (pg ml^−1^) (*n* = 24)**

IFN-γ	22 (5–132)	1804 (15 – 14, 417)
IL-1β	1.5 (<1–5)	45 (< 2.4 – 69)
IL-6	230 (28–2621)	2118 (646 – 12, 928)
IL-8	188 (29–1648)	2066 (578 – 7082)
TNF-α	18 (<1–153)	538 (24 – 3176)
IL-10	3 (<1–9)	73 (< 1.8 – 303)
IL-1ra	758 (85–3833)	2357 (330 – 6295)

Median levels of maternal IL-1β, IFN-γ, TNF-α, and IL-ra were higher during the second trimester. IL-6, IL-8, and IL-10 were higher in the third trimester. For amniotic fluid, median levels for each of the cytokines assessed in this study were higher in the third trimester.

At mid-trimester, IFN-γ and TNF-α were found at higher values in maternal sera compared with amniotic fluid. In this study of samples collected in the third trimester, all the cytokines tested were found at significantly higher levels (*p* < 0.00001) in amniotic fluid compared with maternal plasma (Table 2).

**Table 2:** **Comparison of median cytokine levels (pg ml^−^^1^) in matched samples of maternal plasma, cord blood and amniotic fluid collected from elective cesarean deliveries (*n* = 24)**.

Cytokine	Median (range) (pg ml^−1^)		

	Maternal plasma	Cord blood	Amniotic fluid
Il-6	59 (10–74)	54 (12–3860)	2118 (646–12,928)**
Il-8	90 (36–3289)	95 (23–333)	2066 (578–7082)**
IL-10	11 (<1.8–160)	12 (4–58)	73 (<1.8–303)**
IL1-ra	90 (<4–230)	85 (30–325)	2357 (330–6295)**

**Significant differences between amniotic fluid and cord blood and amniotic fluid and maternal plasma (*p* < 0.00001).

In the mid-trimester samples, IL1-ra levels increased significantly with the age of the mother (10). In this study of samples obtained at term, the only correlation with maternal age was IL-8 (*r* = 0.5789, *n* = 24, *p* = 0.0025, α = 1).

The second objective was to determine if there was a correlation between cytokine levels in maternal or cord blood or amniotic fluid samples obtained during the third trimester. Analyses among the three categories of samples were carried out on the pro-inflammatory IL-6 and IL-8 and the anti-inflammatory IL-10 and IL-1ra.

Levels of each of the cytokines tested were significantly higher in amniotic fluid compared with the matched maternal plasma or cord blood (Table 2). There were no differences in levels of cytokines between maternal plasma and cord blood.

There was a significant correlation between levels of IL-10 in maternal plasma and cord blood (*r* = 0.8506, *n* = 24, *p* = 0.001, α = 2). There were no correlations between levels of any of the cytokines detected in cord blood and amniotic fluid. There was a significant correlation between levels of IL-1ra in maternal plasma and amniotic fluid (*r* = 0.7293, *n* = 24, *p* = 0.001, α = 2).

The third objective was to determine if there were differences in cytokine levels in samples associated with male and female fetuses (Table 3). The levels of the anti-inflammatory IL-1ra were significantly higher in amniotic fluid from female infants (*p* = 0.0009). In amniotic fluid, median IL-1β levels were higher in samples from males (42 pg ml^−1^) compared with those from females (28 pg ml^−1^); however, there were two samples from females and three samples from males in which levels of IL-1β were below the lower limits of detection. There were negative correlations between IL-1β and IL-1ra: for all 24 samples, *r* = −0.296 but these were not significant. The negative correlation was more pronounced for samples from females (*r* = −0.3457) than those from males (*r* = 0.1396). For maternal plasma, there were no significant differences between the levels of cytokines and the sex of the infant. The only significant difference for cord blood was higher median levels of IL-6 for males (70 pg ml^−1^) compared with those for females (39 pg ml^−1^) (*p* < 0.05). IL-1β was measured; however, it was detectable in only four samples of maternal plasma and only one cord blood sample; therefore, it was not analyzed further.

**Table 3:** **Comparison by sex of the infant (males = 12, females = 12) of median cytokine levels in maternal plasma, cord blood, and amniotic fluid obtained during elective cesarean delivery**.

Cytokines	Median (range) (pg ml^−1^)	

	Males	Females
**Maternal plasma**		
Il-6	64 (10–174)	46 (29–170)
Il-8	103 (88–3289)	78 (36–563)
IL-10	10 (<1–15)	13 (4–160)
IL1-ra	76 (4–220)	118 (83–230)
**Cord blood**
Il-6*	70 (32–386)	39 (12–157)
Il-8	99 (59–197)	64 (23–333)
IL-10	10 (4–22)	15 (4–58)
IL1-ra	125 (30–325)	73 (30–293)
**Amniotic fluid**
Il-6	1218 (398–12,928)	2271 (292–10,739)
Il-8	2403 (621–9179)	1794 (578–4186)
IL-10	112 (<1.8–303)	56 (9–226)
IL1-ra**	1839 (330–5065)	3909 (1786–8837)

*p ≤ 0.05.

**p = 0.0009.

Maternal plasma levels of IL-6, IL-8, IL-10, and IL-1ra were assessed in relation to body mass index (BMI) of the mother, birth weight of the infant and GA at birth. For all samples, neither IL-8 nor IL-6 was correlated with any of these three factors. IL-10 was not correlated with BMI or birth weight, but it increased with GA (*r* = 0.3537, *n* = 24, *p* < 0.05, α = 1). IL-1ra was not correlated with birth weight or BMI, but it decreased as GA increased (*r* = −0.5885, *n* = 24, *p* = 0.005, α = 2). For GA, there was a significant association between IL-8 levels with female infants (*r* = 0.8860, *n* = 12, *p* < 0.001, α = 2), and for males, there was a significant negative correlation with IL-1ra levels (*r* = −0.9115, *n* = 12, *p* < 0.001, α = 2).

For cord blood samples, there was no correlation between maternal BMI and any cytokine assessed. For birth weight, there were positive correlations with cord blood IL-6 levels for both females (*r* = 0.666, *n* = 12, *p* < 0.05, α = 2) and males (*r* = 0.5244, *n* = 12, *p* < 0.05, α = 1). For GA, there were no correlations with cytokine levels in cord blood for female fetuses. For males, there was a positive correlation between IL-6 levels and GA (*r* = 0.6819, *n* = 12, *p* < 0.02, α = 2.

Although IL-1ra levels were significantly higher in amniotic fluid of female fetuses, when assessed by the sex of the fetus, the only significant correlation was between GA of male fetuses and IL-1ra (*r* = −0.7225, *n* = 12, *p* = 0.01, α = 1). There was no correlation between birth weight or maternal BMI and levels of cytokines in amniotic fluid of either males or females.

In this study, IL-6 and IL-8 in amniotic fluid rose with GA of the infant; however, only the correlation with IL-6 levels was significant (*r* = 0.3765, *n* = 24, *p* = 0.05, α = 1). Levels of the anti-inflammatory IL-10 and IL-1ra declined with GA, but these were not significant.

## Discussion

The objectives of the study were to determine: if in the third trimester, cytokine levels in amniotic fluid and maternal plasma were higher than those reported for second trimester; if there was a correlation between cytokine levels in the samples obtained from mother and infant; if there were differences in cytokine levels associated with male and female fetuses.

To examine objective 1, data from Chow et al. (10) were compared with our findings as both studies used the same method for detection of cytokines in maternal blood and amniotic fluid. In both studies, IL-6, IL-8, and IL-1ra were detected in all samples of maternal blood. In the mid-trimester blood samples, IFN-γ, IL-1β, and TNF-α were within the range of detection. In the third trimester blood samples, the median levels of these cytokines were lower and many had undetectable levels. In both studies, IFN-γ, IL-6, IL-8, and IL-1ra were detected in all amniotic fluid samples. The median levels of all the cytokines tested (Table 1) were higher in amniotic fluid during the third trimester.

Median cytokine levels in 110 third trimester amniotic fluid samples from cesarean section deliveries not in labor (median age 39 weeks) were lower for IL-6 (764 pg ml^−1^), IL-8 (629 pg ml^−1^), IL-10 (6.6 pg ml^−1^), and TNF-α (10.5 pg ml^−1^) (12) than those reported in the present study. The only significant differences between these cytokine levels and those in samples taken during amniocentesis (median GA 17 weeks) were for IL-10, which was increased and TNF-α which was decreased (12). The higher levels reported in Table 1 are unlikely to be associated with different GAs; the median GA for both studies was 39 weeks. Some of the differences might be attributed to methods for detection of the cytokines; the study by Weissenbacher et al. (12) used an enzyme linked immunosorbent assay.

There were few correlations of cytokine levels between samples from mother and infant. For the samples obtained at mid-trimester, macrophage inflammatory protein (MIP)-1β levels in maternal blood correlated with those in amniotic fluid (10). In this study, levels of IL-1ra in maternal plasma correlated with those in amniotic fluid and maternal IL-10 levels correlated with those in cord blood.

There were no sex-specific differences in cytokine levels in maternal plasma (Table 3). For cord blood, IL-6 was higher in males. For amniotic fluid, most of the pro-inflammatory cytokines, except IL-6, were higher in samples from male fetuses. The major significant difference for amniotic fluid samples was higher levels of IL-1ra in amniotic fluid of female fetuses.

The study of Chow et al. (10) found higher levels of IL-1ra in amniotic fluid compared with those reported by Bry et al. (13); at mid-trimester, there were no sex-specific differences. Our findings agree with those of Bry et al. for third trimester fetuses; the levels for IL-1ra were higher for female fetuses. The correlation that we observed between maternal plasma and amniotic fluid levels of IL-1ra suggests that the pregnant decidua secretes IL-1ra into both amniotic fluid and maternal blood. The relatively high levels of IL-1ra in amniotic fluid compared with maternal blood suggest that IL-1ra is preferentially secreted into the amniotic fluid or that clearance from this compartment is slower than from maternal plasma or a combination of both effects.

It has been proposed that pro-inflammatory cytokines (e.g., IL-6) are important in triggering birth (14, 15). For maternal plasma, both anti-inflammatory cytokines (IL-1ra and IL-10) declined with GA. In pregnancies, where the fetus was male the decrease in IL-1ra with GA was significant, and in male fetuses, there was an increase with GA in the pro-inflammatory cytokine IL-6 in their cord blood. In addition, if the fetus was male, there was a negative correlation between IL-1ra and GA in amniotic fluid. These findings indicate an increasing pro-inflammatory environment as term approaches; and it is most evident in pregnancies in which the fetus is male. IL-1ra levels were higher in the amniotic fluid of female fetuses, suggesting that if the fetus is female not only do levels of the anti-inflammatory cytokine IL-1ra remain constant but also they are significantly higher than those found in amniotic fluid when the fetus is male.

These findings provide preliminary normative data for our population for cytokine levels in late pregnancy and indicate that there are differences in inflammatory responses associated with male and female fetuses. They also indicate that sex of the fetus needs to be assessed in relation to the role of inflammatory responses in the outcome of pregnancy. More work is required to determine the interactions between factors reported to affect inflammatory responses such as higher testosterone levels associated with male fetuses (3, 16) and BMI, which is associated with increased in pro-inflammatory responses (17, 18). It is interesting to note that, in this study, we found that the baby’s birth weight was positively correlated with cord blood levels of the pro-inflammatory cytokine, IL-6. Smoking is associated with decreased anti-inflammatory responses (19). The small number of smokers in the study did not allow us to examine the effect of this important risk factor.

Our results do indicate that female fetuses might be better able to deal with infection and inflammation *in utero* than males and mighty partly explain their lower incidences of pre-term births, stillbirths, and unexpected deaths in infancy. Our data also indicate that term is associated with an increase in inflammatory cytokines and a fall in anti-inflammatory cytokines that is consistent with inflammation playing a role in the onset of human labor. While preliminary, the results are relevant to the highly topical area of sex-specific changes in decidual immune function and the potential effects on both male and female fetuses.

## Author Contributions

Each of the authors made substantial contributions to the conception, design, analyses, and interpretations of the work. They assisted in preparing the article, critically assessed the final version, and agree to be accountable for the accuracy and integrity of the work.

## Conflict of Interest Statement

The authors declare that the research was conducted in the absence of any commercial or financial relationships that could be construed as a potential conflict of interest.
